# Spinocerebellar ataxia 27B (SCA27B)—a systematic review and a case report of a Polish family

**DOI:** 10.1007/s13353-025-00967-3

**Published:** 2025-04-29

**Authors:** Adam S. Hirschfeld, Julia O. Misiorek, Magdalena Dabrowska, Jakub Muszynski, Brandon J. Gerhart, Michał Zenczak, Magdalena Rakoczy, Katarzyna Rolle, Pawel M. Switonski, Jill S. Napierala, Luiza Handschuh, Marek Napierala, Magdalena Badura-Stronka

**Affiliations:** 1 Chair and Department of Medical Genetics, Poznan University of Medical Sciences, Rokietnicka 8, 60 - 806 Poznan, Poland; 2 Diagnostyka GENESIS, J. H. Dabrowskiego 77A, 60 - 406 Poznan, Poland; 3 Department of Molecular Neurooncology, Institute of Bioorganic Chemistry Polish Academy of Sciences, Z. Noskowskiego 12/14 , 61 - 704 Poznan, Poland; 4 Department of Genome Engineering, Institute of Bioorganic Chemistry Polish Academy of Sciences, Z. Noskowskiego 12/14, 61 - 704 Poznan, Poland; 5 Department of Neurology, O’Donnell Brain Institute, University of Texas Southwestern Medical Center, 6000 Harry Hines Blvd., Suite NL9.108 A, Dallas, TX 75390 - 8813 USA; 6 Laboratory of Genomics, Institute of Bioorganic Chemistry Polish Academy of Sciences, Z. Noskowskiego 12/14, 61 - 704 Poznan, Poland; 7 Department of Neuronal Cell Biology, Institute of Bioorganic Chemistry Polish Academy of Sciences, Z. Noskowskiego 12/14, 61 - 704 Poznan, Poland

**Keywords:** LOCA, SCA27B, *FGF14*, GAA repeats, Downbeat nystagmus

## Abstract

Dominantly inherited GAA repeat expansions in the *FGF14* gene have recently been identified as the cause of spinocerebellar ataxia 27B (SCA27B). Our study focused on a Polish patient case along with asymptomatic family members. Moreover, we systematically reviewed available case reports to better understand the SCA27B phenotype. Genetic tests for SCA27B were performed on genomic DNA isolated from blood. Long-range polymerase chain reaction (LR-PCR) followed by Nanopore sequencing was conducted to establish the number of GAA repeats. The available literature was systematically reviewed per the recommendations of the Preferred Reporting Items for Systematic Reviews and Meta-analyses. The patient’s genetic studies identified pure expansions of (GAA) 420/94 repeats in *FGF14,* confirming the SCA27B diagnosis. A systematic review of 815 cases provides further insight into the typical clinical presentation, with gait ataxia (95.96%) being the most prevalent symptom, followed by abnormal saccadic pursuits (80.69%), nystagmus (71.15%), diplopia (54.05%), and dysarthria (51.22%). Notably, 41.87% of cases exhibited episodic symptoms. The correlation between GAA repeat expansions and the pathogenesis of SCA27B requires further studies. The unique course of the disease with episodic symptoms may cause diagnostic difficulties. Due to its high prevalence in the European population, SCA27B should be considered when diagnosing the causes of late-onset cerebellar ataxia.

**Supplementary Information:**

The online version contains supplementary material available at 10.1007/s13353-025-00967-3.

## Introduction

Despite significant progress in next-generation sequencing technologies, approximately half of patients with late-onset cerebellar ataxia (LOCA) remain without a molecular diagnosis (Ngo et al. 2020). Recently, autosomal dominant GAA repeat expansions in the first intron of the fibroblast growth factor 14 (*FGF14*) gene were identified as a frequent cause of LOCA, now classified as spinocerebellar ataxia 27B (SCA27B) (Pellerin et al. 2023). GAA repeats ≥ 300 are considered pathogenic and fully penetrant, and GAA repeats 250–299 are likely pathogenic of reduced penetrance (Méreaux et al. 2024). The prevalence of SCA27B has been reported to range from 1.2% to 61% across LOCA cohorts from diverse ethnic backgrounds. Approximately 15 − 30% of European cohorts of patients with undiagnosed adult-onset ataxia are expected to be affected by SCA27B (Pellerin et al. 2024a). SCA27B is distinguished by specific clinical features that may aid in its diagnosis—including slowly progressive cerebellar ataxia, early episodic symptoms, downbeat nystagmus, and diplopia (Pellerin et al. 2023). 4-aminopyridine (4-AP) is currently being investigated as a potential treatment for SCA27B (Wilke et al. 2023). This work presents the first case of a Polish family affected by SCA27B with a distinctive inheritance pattern. We also systematically review previously reported cases to present detailed clinical phenotypes associated with SCA27B.

## Materials and methods

### Genomic DNA (gDNA)

Samples were procured from the genetic clinic Diagnostyka GENESIS. gDNA was isolated from 400 µL of peripheral blood using an automated extraction process with the dedicated MagCore® Genomic DNA Whole Blood Kit and the MagCore® HF16 Plus Automated Nucleic Acid Extractor (RBC Bioscience).

#### LR-PCR

Amplification of GAA repeat expansions in the *FGF14* gene was performed by long-range PCR using primers FGF14-F: 5'-AGCAATCGTCAGTCAGTGTAAGC and FGF14-R: 5'-CAGTTCCTGCCCACATAGAGC. Reactions were performed in a 50 µL volume with 50 ng of genomic DNA and 1 µM of each primer using the FailSafe PCR System with mix D (Biosearch Technologies) and the following cycling conditions: 80 °C for 3 min, 94 °C for 3 min, then 35 cycles of 95 °C for 10 s, 59 °C for 10 s, and 72 °C for 2 min, followed by a final elongation step of 7 min at 72 °C. To reduce heteroduplex formation, PCR reactions were heated to 94 °C for 4 min and then cooled to 4 °C at a rate of 1 °C/min before electrophoresis. The amplification products were resolved on 1% agarose gels with a 1 kb ladder (Meridian Life Sciences) and stained with SYBR Safe DNA Gel Stain (ApexBio). Gels were visualized using a ChemiDoc MP Imaging System (Bio-Rad). Product sizes were determined using Image Lab software (version 6.1; Bio-Rad), and the number of GAA repeats in each allele was calculated using the equation: [(length of PCR product in base pairs – 165)/3] (Gerhart et al. 2024).

### Nanopore sequencing

LR-PCR products were purified using the QIAquick PCR Purification Kit (Qiagen) and sequenced using Oxford Nanopore technology (Plasmidsaurus). Raw reads in fasta format were analyzed for a consensus sequence. The expanded LR-PCR band from the SCA27B patient was extracted and purified from the agarose gel using the QIAquick Gel Extraction Kit (Qiagen) prior to sequencing.

### Literature review

A systematic review of clinical traits of available SCA27B cases from the PubMed database was performed per the recommendations of Preferred Reporting Items for Systematic Reviews and Meta-Analyzes (Moher et al. 2015). The review protocol is shown in Suppl. Figure 1. Due to the identification of the molecular basis of SCA27B in 2023, we limited the review to a timeframe from Jan 2023 to Jan 2025. We only included reports with detailed descriptions of the clinical features in individual cases or series of cases with definitive clinical signs of SCA27B and genetic diagnoses (GAA repeats ≥ 250).

### Case description

**Fig. 1:**
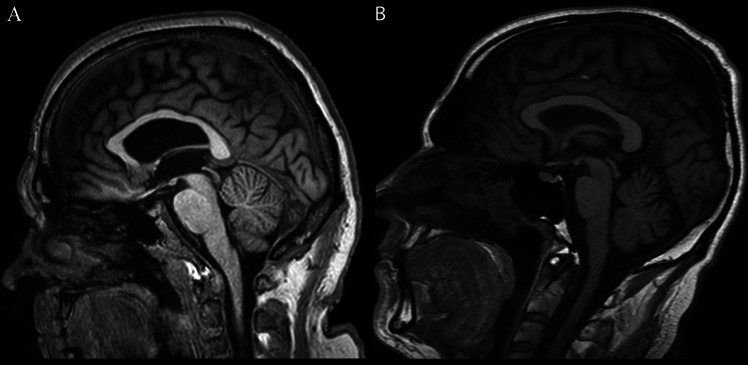
**A**—brain MRI (T1 W sagittal) of a healthy man at the age of 64 y, **B**—brain MRI (T1 W sagittal) of the Polish patient with GAA repeat expansions in FGF14 at the age of 64 y. Slight atrophy of the cerebellum is observed (the primary, secondary, and prepyramidal fissures are open, and a more prominent quadrigeminal cistern is also present)

The patient is a 64-year-old male of Caucasian origin who first developed balance issues at the age of 60 years (y) and progressive bilateral hearing deterioration starting at the age of 50 y. At 60 y, neurological examination showed mild dysarthria, a slight sensory deficit in the sole of the left foot, and mildly impaired coordination in the heel-to-shin test. Otolaryngological examination, including videonystagmography, showed no abnormalities. The initial diagnosis was spinocerebellar ataxia (SCA), considering similar balance disorders observed in the family history (mother, mother’s sister, and grandmother). The patient’s children, a son (41 y) and a daughter (38 y), are asymptomatic. The genetic tests performed excluded SCA types 1, 2, 3, 6, 7, and 8. At age 61 y, the patient was hospitalized in the neurological ward for comprehensive evaluation. Neurological examination revealed ataxic gait with significantly impaired tandem walking. The finger-to-nose test showed left-side dysmetria. Sensory impairment and pallhypesthesia in the distal parts of the lower limbs were detected. Bilateral features of pes cavus were also noted. No disturbances in muscle strength, muscle tone, or deep tendon reflexes were observed. Brain MRI showed cortical atrophy, more pronounced in the cerebellum relative to another healthy man of the same age (Fig. 1).

The neurophysiological examinations indicated a moderate motor neuropathy of axonal type; therefore, Charcot-Marie-Tooth disease was suspected. Whole genome sequencing has found no pathogenic gene variants that could correlate with the patient’s clinical presentation. At age 64 y, the neurological examination showed slightly disturbed gait and mild ataxia of the lower limbs without evident traits of dysarthria or nystagmus. Using the scale for the assessment and rating of ataxia (SARA), the rating was 3 points (Gait—1, Stance—1, Heel-shin slide—1). Additionally, the patient received 4 points on the Friedreich Ataxia Rating Scale-Activities of Daily Living (FARS-ADL). The patient reported that the symptoms have a marked tendency to episodic exacerbation and subsequent remission. Physical exertion may provoke the occurrence of symptoms without aggravation by alcohol and caffeine. Table 1 summarizes the patient’s clinical features. The patient has not been treated with 4-AP or acetazolamide to date.

**Table 1:** Clinical phenotype of the symptomatic proband with GAA repeat expansions in the *FGF14* gene

Disease onset (yo)	60
First symptoms	Impaired gait
Persistent symptoms	Impaired gait, lower limbs ataxia, hearing loss, sensory deficits
Episodic symptoms	Dysarthria, dysphagia, diplopia
Nerve conduction studies	Moderate motor neuropathy of axonal type
Brain MRI	Slight cortical atrophy, more pronounced in the cerebellum
Provoking factors	Physical exertion

## Results

**Fig. 2:**
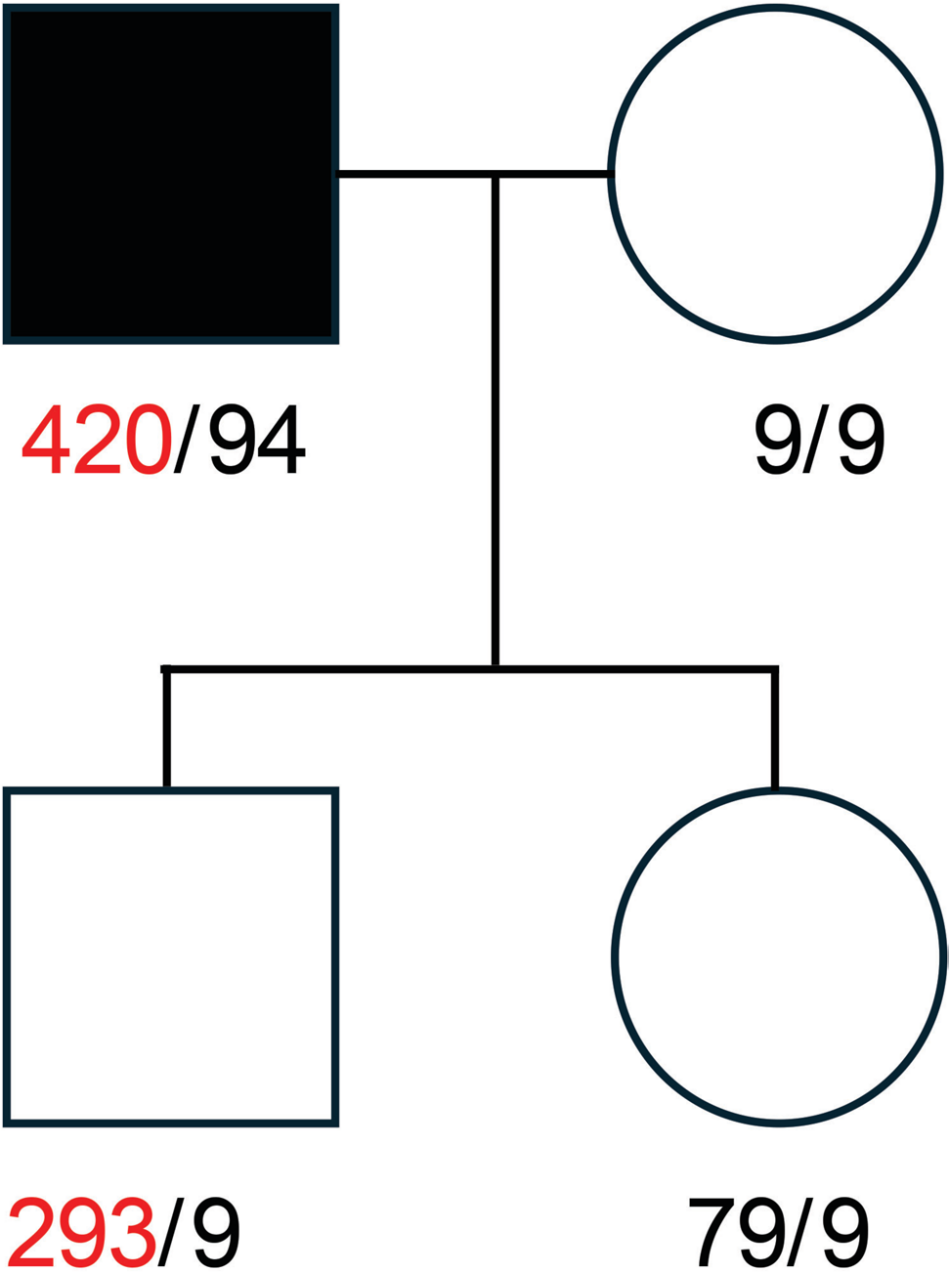
The pedigree of the family affected by SCA27B. Symptomatic proband carries a pathological number of 420 GAA repeats in one allele, which undergoes substantial contraction when transmitted to his son. The shorter paternal allele inherited by the daughter also underwent contraction. The mother is homozygous regarding the GAA repeat number and passes her *FGF14* alleles to the offspring with no change in repeat size. The alleles with GAA expansions are marked in red. The presented GAA repeats number was established by Nanopore sequencing

The patient’s genetic studies identified pure expansions of 420 (GAA) repeats in the expanded allele of *FGF14*, thus confirming SCA27B. The evaluation of GAA repeats in the patient’s asymptomatic children revealed a longer GAA repeat expansion inherited by the son and a shorter allele by his daughter (Suppl. Figure 2). Both alleles contracted upon paternal transmission by 127 GAA units in the son and 15 GAA units in the daughter according to Nanopore sequencing results. The allele with a nonpathogenic repeat length of 9 GAAs from the healthy mother did not change during maternal transmission. The family pedigree with indicated repeat lengths in both *FGF14* alleles is shown in Fig. 2.

Nanopore sequencing of PCR-amplified alleles was performed to confirm the specific amplification of PCR products and determine variants within the 5'-flanking sequence. The results demonstrated the presence of a 5'-common flanking variant (5'-CFV) in both maternal alleles. Both paternal alleles had a 5'-reference flanking sequence (5'-RFS), which differed in one nucleotide. Precisely, thymine was positioned directly upstream of GAA repeats in the expanded allele, while adenine in the shorter allele (Suppl. Tab. 1). This difference enabled correct identification of the parental-inherited allele in the son. Thus, the possibility of inheriting a shorter allele, which expanded upon the transmission to the son, was ruled out.

Based on the review of 30 articles, 815 patients diagnosed with SCA27B were identified (Pellerin et al. 2023; Méreaux et al. 2024; Wilke et al. 2023; Mohren et al. 2024; Satolli et al. 2024; Pellerin et al. 2024c; Seemann et al. 2023; Abou Chaar et al. 2024; Kakumoto et al. 2024; Miyatake et al. 2024; Mendes Ferreira et al. 2024; Pellerin et al. 2024d; Shirai et al. 2023; Lopergolo et al. 2024; Mitrotti et al. 2024; Novis et al. 2023; Rafehi et al. 2023; Livanos et al. 2025; Iruzubieta et al. 2023; Halstuk et al. 2024; Wirth et al. 2023; Foucard et al. 2023; Pinheiro-Barbosa et al. 2024; Kartanou et al. 2024; Bonnet et al. 2023; Ando et al. 2024; Pellerin et al. 2024e; Ouyang et al. 2024; Milovanović et al. 2024). The average onset of symptoms was 57.46 years, with the earliest onset at 6 y and the latest at 80 y (Méreaux et al. 2024; Kartanou et al. 2024). The most common symptoms were gait ataxia (95.96%), appendicular ataxia (80.17%), abnormal saccadic pursuits (80.69%), nystagmus (71.15%) with downbeat nystagmus present in 47.96%, diplopia (54.05%), and dysarthria (51.22%). In 41.87% of cases, some of the symptoms were episodic, with 39.06% of patients reporting an exacerbation of symptoms after alcohol consumption. CNS imaging studies revealed cerebellar atrophy in 70.28% of patients. The available clinical findings of SCA27B patients are summarized in Table 2.

**Table 2:** Clinical phenotype of SCA27B patients. No—number, F—female, M—male, NCS—nerve conduction studies

Cases (No)	815
Sex (F:M)	357/776: 419/776
Mean GAA repeat size	344.09
Disease onset (mean, year of life)	57.46
Disease onset (min–max, year of life)	6–80
Cohort (No)	Australia (16), Brazil (9), Canada (66), China (19), Cypriot (12), France (176), Germany (233), Greece (19), India (3), Italy (70), Israel (2), Japan (28), multicenter (17), Portugal (2), Serbia (9), Spain (18), USA (102)
Impaired/ataxic gait	95.96% (617/643)
Appendicular ataxia	80.17% (275/343)
Upper limb ataxia	56.04% (181/323)
Lower limb ataxia	77.78% (126/162)
Dysarthria	51.22% (378/738)
Dysphagia	34.12% (72/211)
Episodic symptoms	41.87% (296/707)
Alcohol intolerance	39.06% (25/64)
Nystagmus	71.15% (365/513)
Downbeat nystagmus	47.96% (247/515)
Abnormal saccadic pursuits	80.69% (188/233)
Dysmetric saccades	31.96% (31/97)
Diplopia	54.05% (187/346)
Hearing loss	32.35% (44/136)
Hyporeflexia	23.89% (54/226)
Hyperreflexia	33.33% (66/198)
Sensory deficits	36.71% (58/158)
Tremor (any type)	25.34% (150/592)
Abnormal NCS	26.70% (51/191)
Cerebellar atrophy	70.28% (395/562)
Midbrain atrophy	8.18% (13/159)

## Discussion

Experience to date indicates that SCA27B may be one of the most common causes of genetically determined LOCA in specific populations. Of particular note is the European population, where the detectability of this disease in previously unsolved cases seems to be relatively high—France (10.1%) (Méreaux et al. 2024), Germany (23%) (Mohren et al. 2024), and Italy (13.4%) (Satolli et al. 2024). In the German cohort of 320 genetically confirmed ataxia patients, SCA27B accounted for 16% of cases, the second most common diagnosis after SCA3 (19%) (Hengel et al. 2023). Due to geographical proximity, a similar percentage of patients can be expected in the Polish population, and our case indicates that the disease remains undiagnosed in many LOCA patients. Currently, the most frequently diagnosed SCA in the Polish population remains SCA1 (42%) and SCA2 (8%) (Sułek-Piatkowska et al. 2010). SCA27B has several distinctive features. In most patients, the disease seems to progress slower (0.29–0.7 SARA points/year) (Wilke et al. 2023; Satolli et al. 2024) compared to other SCAs—SCA1, SCA3, SCA6 (2.11, 1.56, 0.80 points/year, respectively) (Jacobi et al. 2015) or CANVAS (1.3 points/year) (Traschütz et al. 2021). However, there are several essential aspects to consider regarding assessments using the SARA scale. The evaluation may be influenced by the episodic onset of symptoms. In addition, some symptoms may also undergo episodic intensification at later stages of the disease and/or be triggered by particular factors like alcohol, physical exertion, and caffeine (Ashton et al. 2023). Episodes can range in duration from a few seconds to several days, and most patients develop permanent ataxia, typically within 3–4 years of onset (Ashton et al. 2023). In such cases, the SARA score may be lower due to the absence of certain symptoms during the clinical examination. Moreover, the SARA scale does not evaluate oculomotor symptoms present in many SCA27B cases (e.g., downbeat nystagmus). In the German cohort presenting idiopathic downbeat nystagmus, the (GAA) ≥ 250 expansion was found in 48% (82/170) cases (Pellerin et al. 2024c). The routine evaluation of oculomotor symptoms remains challenging. Patients with LOCA are typically managed by neurologists, who often lack access to essential tools such as video-oculography or videonystagmography, which are needed for standardized assessments of saccades and nystagmus. Lastly, dysarthria does not affect all patients (51.22% in our analyzed cohort), which may reduce the reliability of the evaluation with the SARA scale. Thus, the SARA scale may not be an ideal tool for assessing the course of SCA27B, and other methods should be sought, especially those based on assessing gait impairment, a highly penetrant symptom (95.96% in our analyzed cohort). Attempts have been made to standardize more detailed ways of gait evaluation in SCA27B using motion sensors with promising results (Seemann et al. 2023). This may be of key importance when designing clinical trials, for example, in the case of 4-AP, which shows potential in reducing some disease symptoms. Positive treatment response was observed in two small cohorts, 86% (6/7) (Wilke et al. 2023) and 75% (21/28) (Abou Chaar et al. 2024). Another challenge resulting from the specific course of SCA27B is differential diagnosis. Some patients with polyneuropathy traits (26.70% in our cohort) may be misdiagnosed as late-onset Charcot-Marie-Tooth disease. Detailed data of nerve conduction studies were provided only in some cases—sensorimotor axonal polyneuropathy was present in 22 patients, and sensory polyneuropathy was present in 18 patients. Conversely, SCA27B can be mistakenly identified as cerebellar multiple system atrophy (MSA-C), which has a similar age of onset and can manifest with ataxia and oculomotor symptoms. In one study, 3 out of 24 patients (12.5%) diagnosed with possible MSA-C, based on Gilman’s consensus criteria, were found to have repeat expansions of (GAA) ≥ 250 in *FGF14* (Wirth et al. 2024). SCA27B can be differentiated from MSA-C by positive family history, slower progression, episodic symptoms, and isolated cerebellar atrophy on MRI (if present), while extrapyramidal syndrome, dysautonomia symptoms, and middle cerebellar peduncle atrophy or hyperintensity are more commonly seen in MSA-C (Wirth et al. 2024).

A large percentage of patients with SCA27B analyzed in our cohort (70.28%) demonstrate features of cerebellar atrophy visualized on CNS MRI, which may be an important diagnostic clue. Longitudinal studies indicate that atrophy initially affects the vermis and gradually progresses to the cerebellar hemispheres (Chen et al. 2024). However, in most cases, evaluating cerebellar atrophy relies on the radiologist’s subjective judgment due to the lack of reliable, standardized tools for routine assessment (Baldarçara et al. 2015). Despite identifying expanded GAA repeats in *FGF14* as the cause of SCA27B, the disease requires further investigation. Intergenerational transmission typically leads to an expansion of GAA repeats during maternal transmission and a contraction during paternal transmission when 5’-RFS and pure GAA repeats in the range of 75–250 units are present (Pellerin et al. 2024b). However, the exact mechanism of these changes remains elusive (Pellerin et al. 2024a). Possible incomplete penetrance of alleles containing 250–300 (GAA) repeats and potential factors modifying the course of the disease also remain an open issue (Méreaux et al. 2024). In particular, there are reports of SCA27B occurring with alleles of 234 (GAA) repeats and greater (Hengel et al. 2023). So far, the length of GAA repeats seems to affect the onset and progression of SCA27B moderately compared to Friedreich’s ataxia as another GAA expansion disease (Pellerin et al. 2024a; Long et al. 2017). Implementation of long-read sequencing should improve diagnostic accuracy. Genome-based studies have already revealed a 5'-CFV of GAA repeats, enhancing the stability of repeats almost exclusively in nonpathogenic short alleles (< 30 GAA) (Pellerin et al. 2024b). In the presented family case, the (5'-CFV) is missing on both paternal alleles and is replaced by a 5'-RFS. The presence of both the 5'-RFS and > 250 (GAA) pure repeats was already indicated as modifiers, leading to intergenerational instability and contraction of GAA repeats when passed from the father (Pellerin et al. 2024b). This inheritance pattern was observed in the presented case. Both shorter and longer alleles contracted when passed from the symptomatic father, with a significant 127 (GAA) unit contraction of the longer allele in his son. Such a substantial contraction upon paternal transmission was previously observed in just one out of 123 individuals carrying the 5'-RFS (Pellerin et al. 2024b), where the allele transmitted by the father with pure > 250 (GAA) repeats was shortened by 125 (GAA) units compared to the usual change of 25–50 (GAA) units. At least 17 SCA27B patients were diagnosed with biallelic expansion > 250 GAA—homozygotes or compound heterozygotes (Brais et al. 2023; De et al. 2024; Pellerin et al. 2023; Zeng et al. 2023; Wilke et al. 2023). Detailed clinical data for all these cases were not provided; however, authors reported earlier onset of symptoms (< 30 y) in some patients (Brais et al. 2023; De et al. 2024; Zeng et al. 2023). In one case, the clinical phenotype did not differ from that of heterozygotic patients, and in another, the patient was additionally diagnosed with supranuclear palsy (Wilke et al. 2023).

## Conclusions

SCA27B is becoming increasingly recognized as one of the leading causes of autosomal dominant ataxia in the European population. Given the possible treatment options, distinctive symptoms of this disease should heighten physicians’ awareness when managing patients with unsolved LOCA diagnoses. Additionally, the unique nature of SCA27B necessitates exploring alternative methods for evaluating disease progression and symptom severity beyond the conventional SARA scale. Further molecular and cellular studies are required to understand the mechanistic basis of the disease.

## Supplementary Information

Below is the link to the electronic supplementary material.

Supplementary file1 Supplementary Table 1. Genotypes present in the SCA27B family. The number of GAA repeats in *FGF14* is indicated together with 5'-flanking sequences: either 5'-CFV (common flanking variant), which enhances the stability of repeat locus, or 5'-RFS (reference flanking sequence). Expanded alleles are marked in red. Different nucleotides in paternal 5'-RFS enabling correct identification of inheritance patterns are underlined in the sequence. (PDF 137 KB)

Supplementary file2 Supplementary Fig. 1. The protocol flow chart used during the systematic review. Database used—PubMed. Search query: ((FGF14) OR (SCA27B)) OR (SCA27). (JPG 109 KB)

Supplementary file3 Supplementary Fig. 2. The image of an agarose gel with separated PCR products. The samples of family members are run in lanes 2–5. Control samples of known GAA repeat numbers based on previous Nanopore sequencing were run in parallel (lanes 6–8). 1 kbp marker served as the size reference. Asterisks identify the pathogenic alleles exceeding 250 GAA repeats. (JPG 311 KB)

## Data Availability

All data supporting the findings of this study are available within the paper and its Supplementary Information.
