# Supplementary material for: Spinocerebellar ataxia 27B (SCA27B)—a systematic review and a case report of a Polish family
Source: J Appl Genet. 2025 Apr 29;66(4):895–902. doi: 10.1007/s13353-025-00967-3 (PMC12605495; doi:10.1007/s13353-025-00967-3)
Supplement: Supplementary file 1 — Supplementary file1 Supplementary Table 1. Genotypes present in the SCA27B family. The number of GAA repeats in FGF14 is indicated together with 5'-flanking sequences: either 5'-CFV (common flanking variant), which enhances the stability of repeat locus, or 5'-RFS (reference flanking sequence). Expanded alleles are marked in red. Different nucleotides in paternal 5'-RFS enabling correct identification of inheritance patterns are underlined in the sequence. (PDF 137 KB) [file 13353_2025_967_MOESM1_ESM.pdf]

Family member	Methods applied to estimate the number of GAA repeats in *FGF14*<br>Allele 1 paternal inheritance/Allele 2 maternal inheritance	
	**LR-PCR**	**Nanopore sequencing<br>+ 5'-flanking sequence type**
**Father<br>(symptomatic for SCA27B)**	429/89	420/94<br>5'-RFS CAACCAACTTTCTGA(GAA)$_{420}$<br>5'-RFS CAACCAACTTTCTGT(GAA)$_{94}$
**Mother<br>(healthy homozygote)**	13/13	9/9<br>5'-CFV TAGTCATAGTACCCCAA(GAA)$_{9}$<br>5'-CFV TAGTCATAGTACCCCAA(GAA)$_{9}$
**Son<br>(asymptomatic)**	290/13	293/9<br>5'-RFS CAACCAACTTTCTGA(GAA)$_{293}$<br>5'-CFV TAGTCATAGTACCCCAA(GAA)$_{9}$
**Daughter<br>(asymptomatic)**	72/12	79/9<br>5'-RFS CAACCAACTTTCTGT(GAA)$_{79}$<br>5'-CFV TAGTCATAGTACCCCAA(GAA)$_{9}$

Supplementary Table 1. Genotypes present in the SCA27B family. The number of GAA repeats in *FGF14* is indicated together with 5'-flanking sequences: either 5'-CFV (common flanking variant), which enhances the stability of repeat locus, or 5'-RFS (reference flanking sequence). Expanded alleles are marked in red. Different nucleotides in paternal 5'-RFS enabling correct identification of inheritance patterns are underlined in the sequence.
