# Supplementary figures and images for: Spinocerebellar ataxia 27B (SCA27B)—a systematic review and a case report of a Polish family
Source: J Appl Genet. 2025 Apr 29;66(4):895–902. doi: 10.1007/s13353-025-00967-3 (PMC12605495; doi:10.1007/s13353-025-00967-3)

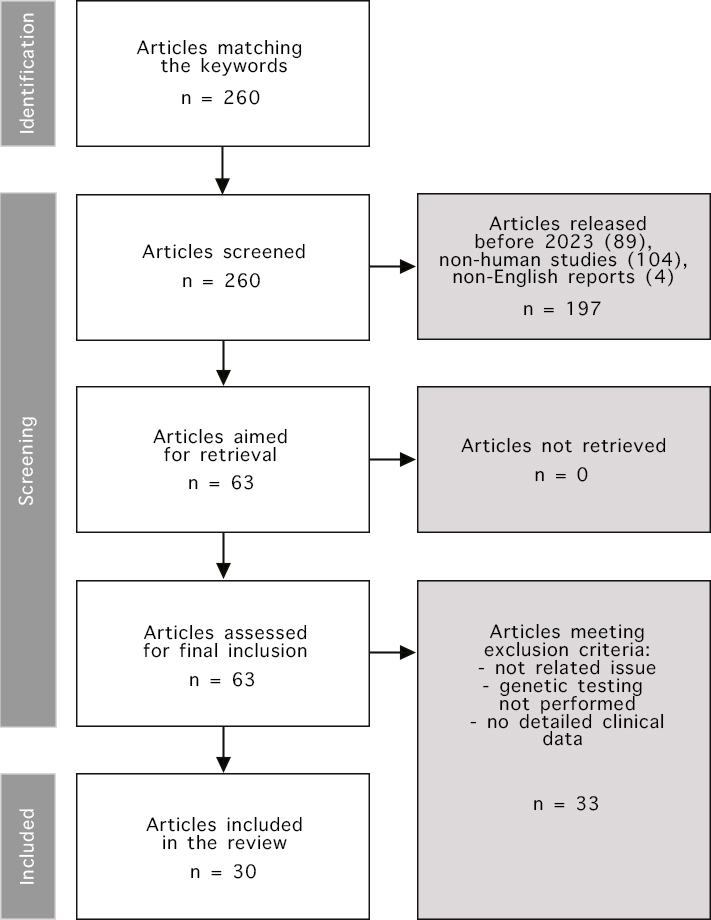

Supplement: Supplementary file 2 — Supplementary file2 Supplementary Fig. 1. The protocol flow chart used during the systematic review. Database used—PubMed. Search query: ((FGF14) OR (SCA27B)) OR (SCA27). (JPG 109 KB) [file 13353_2025_967_MOESM2_ESM.jpg]

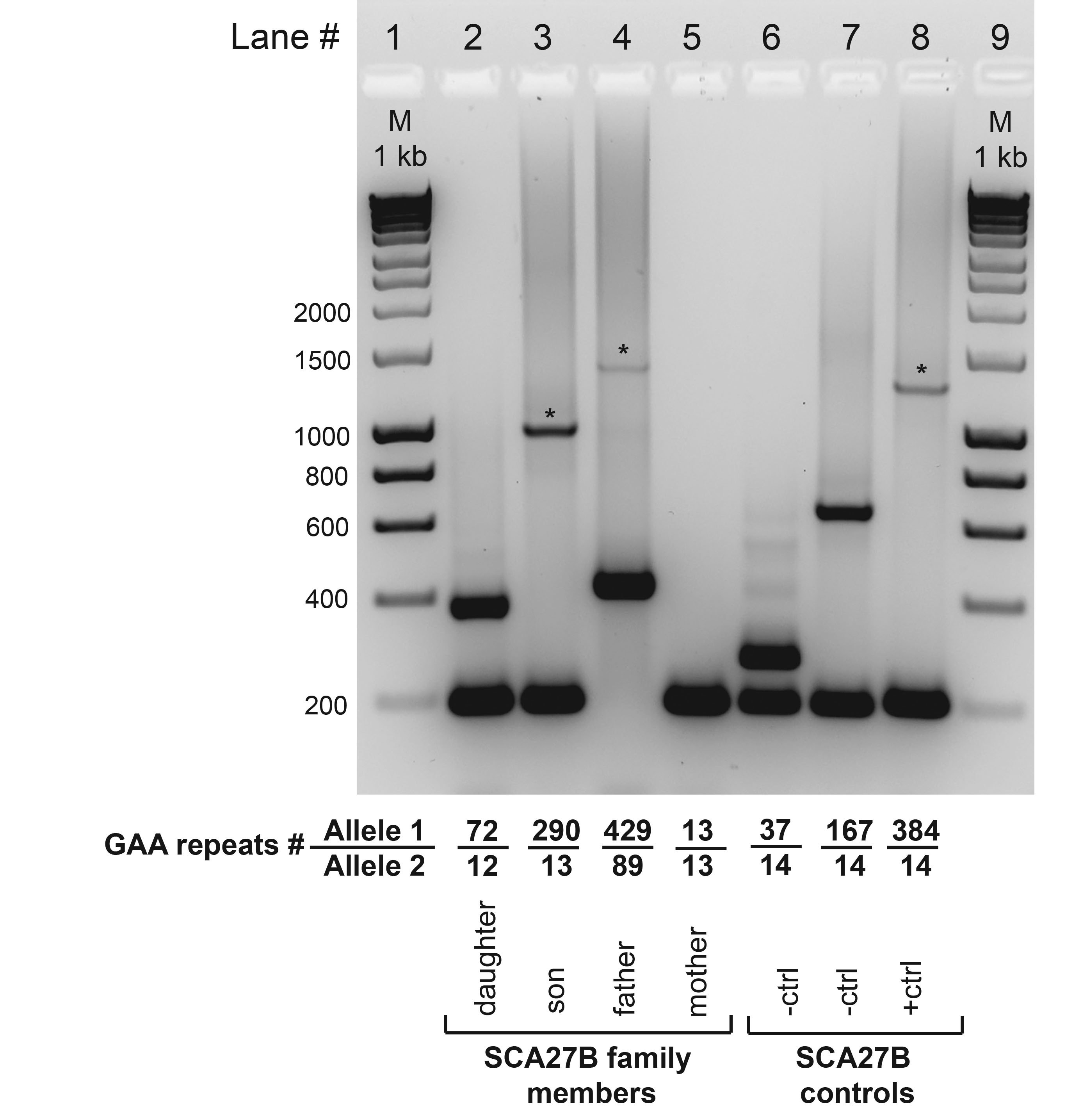

Supplement: Supplementary file 3 — Supplementary file3 Supplementary Fig. 2. The image of an agarose gel with separated PCR products. The samples of family members are run in lanes 2–5. Control samples of known GAA repeat numbers based on previous Nanopore sequencing were run in parallel (lanes 6–8). 1 kbp marker served as the size reference. Asterisks identify the pathogenic alleles exceeding 250 GAA repeats. (JPG 311 KB) [file 13353_2025_967_MOESM3_ESM.jpg]
